# Trust in Science, Perceived Media Exaggeration About COVID-19, and Social Distancing Behavior

**DOI:** 10.3389/fpubh.2021.670485

**Published:** 2021-12-01

**Authors:** Ariadne Neureiter, Marlis Stubenvoll, Ruta Kaskeleviciute, Jörg Matthes

**Affiliations:** Department of Communication, University of Vienna, Vienna, Austria

**Keywords:** perceived media exaggeration about COVID-19, trust in science, social distancing behavior, panel study, COVID-19

## Abstract

For many individuals, the media function as a primary source of information about preventative measures to combat COVID-19. However, a considerable number of citizens believe that the media coverage about pandemics is exaggerated. Although the perception of media exaggeration may be highly consequential for individual health behaviors, we lack research on the drivers and consequences of this perception. In a two-wave panel study, we examined associations between trust in science, perceptions of media exaggeration about COVID-19, and social distancing behavior during the lockdown in Austria (*N*_T2_ = 416). Results showed that trust in science at T1 led to less perceptions of media exaggeration about COVID-19 at T2. Furthermore, consistent with the theory of psychological reactance, perceptions of media exaggeration about COVID-19 at T1 caused less social distancing behavior at T2. Thus, findings suggest that trust in science may positively affect individuals' social distancing behavior by decreasing perceived media exaggeration about COVID-19 over time. Implications for research on media effects in times of COVID-19 and conclusions for journalists are discussed.

## Introduction

At the beginning of 2020, scientific experts and governments urged citizens to change their social behavior by implementing social distancing practices in order to stop an exponential spread of the virus SARS-CoV-2 (1). However, there is an increasing concern that people do not comply with the proposed preventative measures (2–5). The disregard of social distancing practices could lead to an uncontrolled spread of SARS-CoV-2 and subsequently to many deaths due to COVID-19 [(2), p. 2]. Governments and health experts increasingly recognized that compliance of the public with preventative health measures is essential in order to overcome the virus by stopping its uncontrolled spread (2). However, little is known about contributing factors to people's adherence to preventative practices regarding COVID-19 (2, 6). As the media^1^ play an essential role in health crises—because it functions as a primary source of information about pandemics for many people—this paper seeks to shed light on media related factors contributing to compliance with social distancing policies (7, 8).

However, media may be also perceived by the public as exaggerating facts about the unknown virus and portraying worst-case scenarios when reporting about epidemics and pandemics (7, 9–11). As a result, many individuals perceive media coverage in these times as too intensive and as exaggerated in regard to health crises (12–14). This is alarming, since media exaggeration due to threatful media coverage in the context of COVID-19 could lead to opposite behaviors than intended by health planners and government (14, 15). Drawing on the theory of psychological reactance (16), we theorize that perceptions that the media are exaggerating about COVID-19 may result in lower compliance with preventative health behavior such as social distancing practices (7, 13, 15).

Nevertheless, as individuals hold a rather high level of trust in science (17), it has been commonly assumed that scientific sources may have a positive influence on the audiences' perception of the media coverage about COVID-19 due to mental shortcuts in information processing (18). In the context of COVID-19, scientists are included in media coverage on a daily basis, educating the audience about the unknown virus and supporting the government in establishing new policies (11, 19, 20). Therefore, trust in science could be a factor contributing to a more positive perception of media coverage about COVID-19, and hence, reduce the perceptions of media exaggeration (21).

In this article, we tested for the first time whether (1) trust in science can contribute to less perceived media exaggeration about COVID-19, and whether (2) perceived media exaggeration about COVID-19 has an influence on social distancing behavior over time. For this purpose, we conducted a two-wave panel study with a one-month-interval in times of the lockdown in Austria due to the COVID-19 outbreak.

### Media Coverage in Times of Covid-19

In times of health crises, media usage is increasing exponentially (22). Previous studies have shown that during the SARS outbreak in 2003, the majority of individuals have used media to inform themselves about risks and measures to combat the virus (23, 24). Similarly, during the outbreak of the swine flu in 2009, individuals have relied on media as their primary source of information about possible health risks and preventative methods to avoid getting infected with the swine flu (25).

Since citizens are highly dependent on media in crisis situations, the media bear responsibility for reporting informatively and adequately (26). However, in the context of health crises, media can be described as a ‘double-edged sword' [(7), p. 44]. On the one hand, media educate citizens about risks and new developments by providing new information about the virus (7). On the other hand, the public may perceive media coverage as overstating health risks due to sensationalism and panic-inducing elements in the reporting on the virus (7, 10, 11, 14, 26–28). Moreover, previous research in the context of the COVID-19 outbreak suggests that the media rather use language inducing “scaremongering” than language promoting self-efficacy [(14), p. 265]. In line with this finding, a previous content analysis of the news articles during the avian flu has shown that over 40% of the articles reporting about the pandemic in U.S. newspapers included worst-case scenarios (9). Similarly, media portrayed the SARS virus and the swine flu mostly in terms of risks using a strong and alarming language instead of prevention language elements (12, 29). Collectively, these studies outline the intensive and alarming character of media coverage during health crises (11, 12).

Individuals past experiences with such potentially exaggerating media messages in health crises might have severe consequences. One consequence of false or exaggerated alarms about health crises in the past is a possible desensitization of the public to real threats. As stated by Bennett, an “incessant ringing of alarms about dubious problems, unseemly scandals, and daily threats to health and safety discourages citizens from taking the press, politicians, and public life seriously” [(30), p. 131]. If a real threat arises, for instance, the threat of overburdening the health care system in the case of a rapid spread of COVID-19, the perception of exaggeration might arise and impede media's function of informing the public and transmitting important messages from health officials. Against this backdrop, it is important to find ways to effectively mitigate this perception of media exaggeration in the case of COVID-19 reporting. In this study, we specifically investigate the role of trust in science in reducing harmful perceptions of media exaggeration. We define trust in science as individuals' trust in scientists to create unbiased knowledge, inform the public, and give advice on policies (31).

### Trust in Times of Covid-19

Generally, in times of COVID-19, people tend to “rally around the flag” reporting stronger trust in politicians and more satisfaction with the government than before the COVID-19 crisis [e.g., (32)]. Additionally, satisfaction with healthcare has not worsened in the course of the pandemic (33). Despite the rise of scientific uncertainty, misinformation, and conspiracy theories during COVID-19 (34), trust in science has increased during the COVID-19 pandemic (33) which decreases the susceptibility to COVID-19 related misinformation that, in turn, could negatively influence compliance with public health measures (35). In this context, previous research has drawn attention to the importance of risk communication timing in the COVID-19 crisis. Drawing on this research, extreme beliefs (like conspiracy beliefs) could be mitigated by early risk communication using scientists as a trusted source of information (36).

More generally, in health crises, trust in different actors is seen as crucial for combating them (37). While actual threat of the virus does not predict compliance with social distancing measures in times of COVID-19, trust toward fellow citizens, the media, government and science do (38–40). For instance, several studies have shown that trust in the government is necessary for compliance with (health) measures [e.g., (34, 41)]. One study showed that in European regions where prior to the COVID-19 crisis trust in policy makers was high, people have restricted their mobility to a higher extent during the COVID-19 crisis than in regions without high trust in policy makers (37). Hence, it is assumed that political trust positively influenced social distancing measures. However, at the same time, there is evidence that trust in government is dependent from individuals' so-called “moral foundations” [(39), p. 9]. When individualizing foundations (i.e., care and fairness) are endorsed stronger than binding foundations (i.e., loyalty and authority), trust in science is stronger for these individuals than trust in the government (39). Both, trust in government as well as trust in science are important factors in combating the pandemic. However, they could lead to different outcomes. More precisely, the more governmental trust people have, the less they perceive the crisis as a risk or threat. Interestingly, the relationship between trust in science and risk perceptions is reverse: the more trust in science, the more risks are perceived (41). Although risk perceptions are obviously an important factor when looking at trust in science, this paper focuses on the influence of trust in science on perceived media exaggeration and the consequences on social distancing behavior. Risk perceptions are likely to influence these variables too, but this goes beyond the scope of this paper. However, it is discussed in the discussion section.

### Trust in Science and Perceived Media Exaggeration

In health crises, scientists are a frequent source of statements and messages about preventative health measures in the media (2, 11). Since the beginning of the COVID-19 crisis, the media made references to science and scientific experts on a daily basis (1), supporting politicians in explaining the necessity and measures to combat COVID-19 (20). More precisely, when looking at the presence of scientists (like geneticists, sociologists, and psychologists), expert opinions on COVID-19 were included in newspaper reporting on COVID-19 most often in the beginning of the pandemic when many countries introduced a lockdown [March, April and May 2020; (42)]. Scientists not only inform and consult policy makers and the public on how to find solutions and manage health crises, but their opinions and attitudes were often included in media reporting. They play an important role in the development of a health crisis as their attitudes toward policy tools can influence the government in their decisions about measures to combat the crisis as well as the public's opinion toward these measures (34).

It is important to note that scientists sometimes have different risk perceptions, opinions and views on policy tools and measures depending on their research field [e.g., (43, 44)]. For instance, Aranzales et al. (43) showed that health scientists and social scientists have different attitudes toward immunity certificates as an instrument to contain the spread of the coronavirus. Nevertheless, in the early days of the pandemic, mostly virologists and epidemiologists were visible in media reporting. This overreliance on a rather small number of expert voices generated a picture of unison around the necessity of drastic health protective measures, which has also been criticized by scholars (45).

Due to high levels of trust in science in the general population (17), researchers observed increasing “attention and information-seeking” of scientific information by the general public since the COVID-19 outbreak [(46), p. 15]. Especially in uncertain situations such as the outbreak of an unknown virus like SARS-CoV-2, trust in science and in experts is very important as people lack the knowledge to understand the health risks of the new virus (46, 47). Reliance on trusted sources is visible also in other contexts, such as individuals' voting behavior, where research has shown that high levels of complexity lead to increased reliance on trusted representatives in voting decisions (48). Thus, taking into account the unknown and complex nature of the pandemic especially at its beginning, individuals may be more ready to rely on and trust the scientists.

Traditional media outlets, first and foremost the public broadcaster, were the primary source of scientific information in the early days of the pandemic in Austria: 62% of the population indicated to use the public broadcaster on a daily basis to get information about COVID-19 (49). Content analytical evidence showed that legacy media reporting was flooded with content that predominantly transmitted information about current policies as well as scientific evidence and statistics during the first lockdown (50). As some scholars have criticized, this period of reporting was also marked by a high level of “announcement journalism,” where media outlets directly and uncritically reported scientists' views as facts (45). This has led to a mainly uncontested transmission of scientific evidence to the public via the legacy media.

In this unique situation, in which politics, media, and scientists became closely entangled in communicating the threat of COVID-19, changes in trust in science are likely to not only affect how individuals react to scientists directly. They might additionally exhibit spill-over effects to the primary messenger, that is, traditional media. Specifically, trust in science might influence perceptions on media reporting via two main paths: The interdependence of trust in institutions and micro-level effects stemming from individuals' information processing. As a result, individuals may perceive media exaggeration. Throughout the paper, the term perceived media exaggeration is used to refer to individuals' perceptions that the media inflates the crisis by portraying the virus as more dangerous than it really is. Drawing on previous research indicating that scientific messages were dominant in the media in times of COVID-19, these perceptions contain, besides general content, also scientific content (45, 50).

Previous research has established that trust in different institutions is highly interlinked. Especially trust in legacy media can be consistently predicted by individuals' broader evaluations of democratic institutions, forming a so-called “trust-nexus” (51). As argued by Earl Bennett et al. (52), institutions might “rise and fall together” (p. 18), reflecting larger societal trends of trust in institutions and authorities. One possible explanation for the observed spill-over from the evaluation of other institutions to the evaluation of media lies in individuals' generalizations about “the elites,” for example in populist discourses [(51), see also (53)]. Citizens can form generalized ideas about elites, and therefore the behavior and evaluation of one elite actor group might also positively or negatively reflect on other actors assigned to this category.

Second, trust in science might affect perceptions of media exaggeration on the level of information processing of individual messages. Specifically, perceived source expertise and coherence have been established as important factors which explain how individuals evaluate and act upon new information (21).

In regard to source expertise, a considerable amount of literature suggests that the message source influences the persuasiveness of a message and how people perceive a message (54). Therefore, when trusted communicators such as scientists share their expertise in the media by either communicating themselves about it or through journalists, this might have positive consequences for individuals' overall perceptions of the COVID-19 coverage. This can be explained through people's use of mental shortcuts, so-called cues, in information processing (18). Authority cues, such as a source's background, profession, or title, represent a specific form of mental shortcut, through which individuals are able to determine quickly and with low levels of cognitive effort that a certain message is trustworthy (21). When individuals place trust in scientists, whose expertise is given ample room in the COVID-19 media coverage (1), the effect of authority cues might be more pronounced and may positively spill over to their evaluation of message credibility.

This, in turn, might reduce perceptions of media exaggeration. Paralleling these results, research has also shown that those who hold negative attitudes toward experts react negatively to such expert cues and might even strengthen their opposition to the message (55). Thus, in a situation where expert cues and scientific voices are predominant in the media (45, 50), individuals low in trust in science might evaluate the coverage on COVID-19 as exaggerating.

Second, even without a direct presence of scientific voices in the media coverage, trust in science might affect perceptions of media exaggeration. Scientists and scientific bodies have strongly urged the public to take the threat of COVID-19 seriously and asked citizens to act in accordance with social distancing rules (56). When messages similar to those of trusted scientists are found in the media, individuals who place greater trust in scientists might perceive more alignment between their own, scientifically colored interpretations of the COVID-19 situation and the messages in media outlets, which have overall highlighted the severity of the virus (45, 50). Thus, individuals with greater trust in science might perceive a higher co-orientation or congruency between their own views and those of media outlets when they report about the threat of COVID-19 (57). Co-orientation, an idea grounded in balance theory (58), refers to “the similarity between one person's cognition about an object and estimate of another person's cognition about that object” [(57), p. 103] and has been found to be a driving factor of message and messenger credibility.

Thus, it seems plausible to expect that trust in science reduces perceived media exaggeration about COVID-19 over time. Hence, we derive our first hypothesis.

H1: Trust in science decreases perceived media exaggeration about COVID-19 over time.

### Decreased Social Distancing Behavior as a Result of Perceived Media Exaggeration

At the end of March/beginning of April 2020, the government introduced a lockdown in Austria including various measures to combat COVID-19. Schools and universities closed, events were postponed, and parts of Austria were quarantined. Moreover, people were urged to stay at home, comply with social distancing measures, and wear face masks. Until today, social distancing is communicated as the most important measure by health officials.

Social distancing behavior refers to the reduction of ones' social interactions to a minimum (59). Social distancing has been introduced by a number of governments worldwide as non-pharmacological measure to flatten the infection curve of COVID-19 (60). By introducing social distancing measures, governments urge individuals to maintain physical distance to other people, avoid crowds, and spend most time of the day at home (59, 61). Whereas previous research has shown that social distancing behavior negatively contributes to individuals' mental health by increasing stress, depression, and insomnia (62), it has also been demonstrated that social distancing behavior has the potential to reduce deaths due to COVID-19 (60).

The introduction of social distancing and other measures was accompanied by extensive media coverage (50, 63). Traditional media outlets served as a main channel for politicians to guide public behavior and keep citizens informed about the necessity of these measures (64). The positive role of traditional media is also reflected in fewer misperceptions and higher levels of social distancing compliance among its users (65).

Increasing individuals' trust in science and subsequently reducing perceptions of media exaggeration could therefore have positive consequences for social distancing behavior. Recent evidence in the context of the COVID-19 pandemic suggests that individuals' perceptions of how the media are reporting on COVID-19 determines people's behavior regarding the virus (66). Not only how much attention the media pay to COVID-19 topics, but also how the media frame the epidemic influences individuals' intended health behavior (14, 67). In the context of the H1N1 pandemic, research identified the use of sensationalized and alarmist media frames (25, 67). Similarly, some first content analytical findings suggested that media used alarmist tones, such as words “deadly disease” or “scary,” when reporting about COVID-19 at the beginning of the outbreak (68). Thus, media may report in an alarming way especially at the beginning of the pandemic which is typically surrounded by uncertainty and the lack of factual information. Nonetheless, in addition to the amount, the tone and framing of messages, importantly, also individuals' evaluations of the reporting such as the perceived media exaggeration about COVID-19 could have serious implications for their social distancing behavior.

Drawing on the Theory of Psychological Reactance (16), alarming messages which are perceived as threatening may evoke psychological reactance against these messages (69). Reactance can be described as a motivational state which arises when a certain freedom is under threat. In the context of health communication, this threat is perceived as especially pronounced when a message employs forceful language (70). In other words, the more a message pushes an agenda and the less it leaves individuals room to hold a dissenting opinion, the more people feel threatened and perceive a need to restore their freedom. In the context of COVID-19 communication, the perception of media exaggeration can be understood as a direct judgement of the unjustified forcefulness of a claim (71). Thus, there is a high likelihood that individuals' perceptions of media exaggeration evoke reactance.

When feelings of reactance are experienced, attempts to mitigate these feelings will emerge. The performance of the threatened behavior is seen as the most basic form of mitigation of feelings of reactance (16). Moreover, it has been empirically shown that individuals' perceptions that the media exaggerate health risks gave rise to non-compliance with preventative measures to reduce these risks (13, 72). In the case of the COVID-19 health crisis, we argue that individuals' perceptions that the media exaggerate about COVID-19 weaken the social distancing behavior due to psychological reactance processes.

We thus suggest our second hypothesis.

H2: Perceived media exaggeration about COVID-19 decreases social distancing behavior over time.

## Method

### Sampling and Procedure

Between March/beginning of April 2020 (= T1) and May 2020 (= T2), we conducted a two-wave panel survey with a one-month interval. The survey was carried out during the lockdown due to the COVID-19 outbreak in Austria. We have chosen a one-month interval for data collection, because we wanted to make sure that in the period between our two waves, no other events take place that could intervene with our measures from wave one (73). Therefore, we have chosen a timeframe that falls within the period of the first lockdown in Austria. At the time of the first wave, the Austrian government imposed the lockdown and urged people in Austria to leave their homes only when they go to work, for doctor's appointments, for basic care, assistance for persons in need, and outdoor exercise. Otherwise, the government asked Austrians to stay at home and to reduce social contacts (74). All shops except for supermarkets and pharmacies, as well as schools, were closed. At the time of the second wave, the lockdown had officially ended, but most measures were still in effect. For instance, schools and restaurants only reopened after the second wave of data collection (75). Besides leisure activities and community life, mental health and quality of life was negatively impacted by these restrictions due to COVID-19 (76–78). All in all, in Austria, during our data collection the daily average of people infected with COVID-19 was 224, the daily average of people in intensive care due to COVID-19 was 120, and the daily average of deaths due to COVID-19 was 11 [means calculated for first and second wave of data collection; (79)].

Our sample was collected by the professional online polling institute Dynata based on representative quotes for age, gender, and educational level in Austria, and a complementary, simultaneous sample collected at the University of Vienna using the same questionnaire, methodology, and quota plan. In order to participate, subjects had to provide consent, indicate that they are using a smartphone, and must at least have reached 16 years of age.^2^

In the first wave (T1), a total of *N* = 731 participants (*M*_*age*_ = 40.49, *SD*_*age*_ = 13.33; 53.9% women; 20.5% no education and lower-secondary education, 46.5% secondary education, 33.0% complete University education) completed the survey. In the second wave (T2), a total of *N* = 416 participants (*M*_*age*_ = 41.97, *SD*_*age*_ = 13.59; 54.3% women; 21.6% no education and lower-secondary education, 45.0% vocational school education and secondary education, 33.4% complete university education) participated.

A total of 43.1% of participants who took part in the survey at T1 dropped out at T2. There was no significant difference between participants who dropped out after the first wave (T1; *n* = 315) and participants who also completed the second wave (T2; *n* = 416) regarding gender [χ^2^(2) = 1.37, *p* = 0.504], education [χ^2^(5) = 5.33, *p* = 0.377], trust in the government [*t*_(729)_ = 0.49, *p* = 0.623], perceived media exaggeration [*t*_(729)_ = −0.80, *p* = 0.422], and social distancing behavior [*t*_(729)_ = 0.55, *p* = 0.584]. Participants who dropped out at T2 indicated a lower age (*M* = 38.53, *SD* = 12.80), and less trust in science (*M* = 3.67; *SD* = 0.87) than respondents who participated in both surveys [age: *M* = 41.98, *SD* = 13.55, *t*_(729)_ = 3.49, *p* = 0.001; trust in science: *M* = 3.86, *SD* = 0.88, *t*_(729)_ = 2.93, *p* = 0.003]. Prior to commencing the study, we sought ethical clearance from the Institutional Review Board of the Department of Communication, University of Vienna.

### Measures

#### Trust in Science

We assessed *trust in science* with three items derived from McCright et al. (31) on a five-point Likert scale ranging from “completely distrust” to “completely trust.” We asked participants to indicate how much they trust scientists and researchers to create knowledge that is unbiased and accurate, to inform the public on important issues, and to advise government officials on policies (T1: Cronbach's α = 0.89; *M* = 3.77, *SD* = 0.88; T2: Cronbach's α = 0.89; *M* = 3.57, *SD* = 0.89).

#### Perceived Media Exaggeration About COVID-19

We measured *perceived media exaggeration about COVID-19* with the following three customized items on a five-point Likert scale ranging from “strongly disagree” to “strongly agree”: “The media unnecessarily inflate the corona crisis,” “The media are unnecessarily scaring people about the coronavirus,” “The media portray the coronavirus situation as worse than it really is” (T1: Cronbach's α = 0.91; *M* = 2.51, *SD* = 1.05; T2: Cronbach's α = 0.92; *M* = 2.75, *SD* = 1.06).

#### Social Distancing Behavior

For *social distancing behavior*, we used three customized items. We asked participants to indicate their agreement to the following three statements on a on a five-point Likert scale ranging from “strongly disagree” to “strongly agree”: “When I go outside, I try to avoid contact with other people as much as possible,” “When I meet other people outside, I keep about 2 m distance to them,” “I try not to talk to other people when I leave my apartment” (T1: Cronbach's α = 0.59; *M* = 3.66, *SD* = 0.99; T2: Cronbach's α = 0.64; *M* = 2.85, *SD* = 1.05).

#### Controls

As control variables, we asked for participant's age, gender, education, political orientation, trust in the government, and quality and tabloid media use. Moreover, we controlled for sampling type (0 = online Dynata quota sample, *N* = 164; 1 = online quota sample, *N* = 252).

### Data Analysis

For data analysis, we conducted Structural Equation Modeling with Full Maximum Likelihood estimation using SPSS Amos (80). We controlled for autoregressive effects (i.e., trust in science at T1 as a predictor for trust in science at T2). We tested for longitudinal measurement invariance of all latent variables (81) by constraining the factor loadings of all the latent variables at T1 and T2 to be equal. The model fit of the constrained model is good: CFI = 0.97; TLI = 0.96; NFI = 0.94; χ^2^/df = 1.77, *p* < 0.001; RMSEA = 0.03, 90%-CI [0.03; 0.04]. No statistically significant difference between the unconstrained and the constrained model was found [χ^2^(6) = 10.76, *p* = 0.096]. Thus, the constructs show metrical invariance over time.

## Results

**Figure 1:**
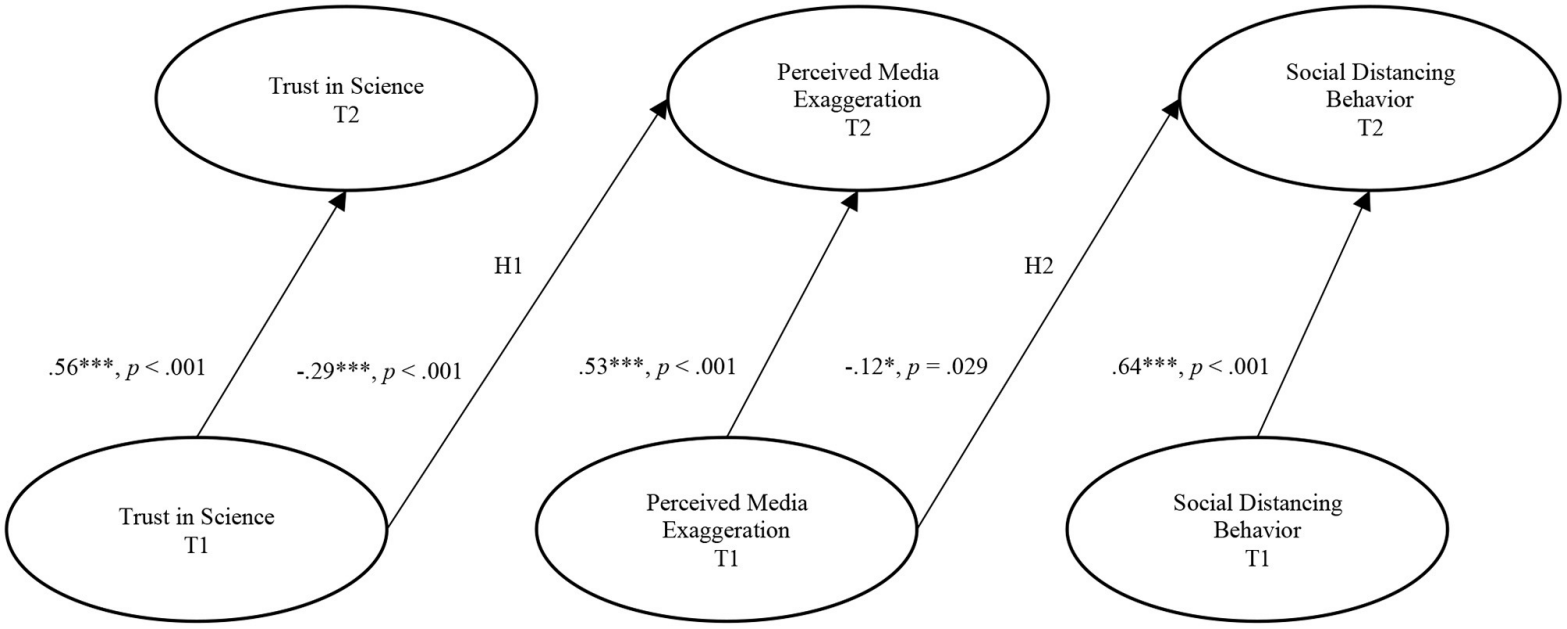
Model considering the relationships between trust in science, perceived media exaggeration, and social distancing behavior. Values represent unstandardized coefficients. Ovals represent latent variables. Error terms, covariances, control variables, and measurement items are not shown. T1, Time 1; T2, Time 2. *p < 0.05, ***p < 0.001.

Pearson correlations are depicted in Table 1. The main results are presented in Figure 1 and the results of the structural model are shown in Table 2. Table 3 shows means and standard deviations of variables included in the SEM model.

**Table 1:** Pearson correlations.

	**1**.	**2**.	**3**.	**4**.	**5**.	**6**.	**7**.	**8**.	**9**.	**10**.	**11**.	**12**.	**13**.	**14**.	**15**.
1. Age	1														
2. Gender (female)	−0.13^**^	1													
3. Education (low)	0.15^**^	−0.11^**^	1												
4. Education (high)	−0.11^**^	0.15^***^	−0.36^**^	1											
5. Left-right orientation	0.12^*^	−0.06	0.17^**^	−0.17^**^	1										
6. Use quality media	−0.02	−0.01	−0.21^**^	0.19^**^	−0.15^**^	1									
7. Use tabloid media	0.21^**^	−0.08^*^	0.18^**^	−0.21^**^	0.26^**^	0.02	1								
8. Trust in the government	0.05	0.11^**^	−0.05	0.05	0.06	0.04	0.02	1							
9. Sampling type	−0.06	0.03	−0.21^**^	0.24^**^	−0.19^**^	0.19^**^	−0.25^**^	0.05	1						
10. Trust in Science (T1)	−0.01	0.06	−0.10^**^	0.15^**^	−0.18^**^	0.13^**^	−0.06	0.55^**^	0.10^**^	1					
11. Trust in science (T2)	0.05	0.06	−0.12^*^	0.14^**^	−0.14^*^	0.16^**^	−0.06	0.48^**^	0.21^**^	0.62^**^	1				
12. Perceived media exaggeration (T1)	−0.04	−0.08^*^	0.08^*^	−0.13^**^	0.15^*^	−0.11^**^	0.08^*^	−0.39^**^	−0.06	−0.45^**^	−0.39^**^	1			
13. Perceived media exaggeration (T2)	−0.03	−0.12^*^	0.11^*^	−0.18^**^	0.22^**^	−0.12^*^	0.14^**^	−0.37^**^	−0.19^**^	−0.45^**^	−0.58^**^	0.64^**^	1		
14. Social distancing behavior (T1)	−0.12^**^	0.13^**^	−0.10^*^	0.09^*^	0.00	0.06	−0.03	0.21^**^	0.01	0.20^**^	0.14^**^	−0.17^**^	−0.11^*^	1	
15. Social distancing behavior (T2)	−0.11^**^	0.05	−0.07	0.03	−0.05	0.09	0.04	0.15^**^	0.10^*^	0.16^**^	0.18^**^	−0.18^**^	−0.23^**^	0.45^**^	1

*N_T1_ = 731, N_T2_ = 416; T1 = Time 1, T2 = Time 2*.

* *p < 0.05*,

** *p < 0.01*,

*** *p < 0.001*.

**Table 2:** Results of the structural equation model.

**Predictor**	**Trust in science (T2)**		**Perceived media exaggeration**		**Social distancing behavior (T2)**	
			**about COVID-19 (T2)**			
	***b***	***SE***	***b***	***SE***	***b***	***SE***
Age	0.01	0.00	−0.00	0.00	−0.00	0.00
Gender (female)^a^	−0.03	0.06	0.03	0.09	−0.04	0.10
Education (low)^a^	−0.11	0.08	0.09	0.12	−0.13	0.13
Education (high)^a^	−0.05	0.07	−0.08	0.10	−0.11	0.12
Sample type (online quota sample)^a^	0.23^**^	0.07	−0.23^*^	0.10	−0.27^*^	0.11
Left-right orientation	−0.00	0.02	0.04	0.03	−0.03	0.03
Use quality media	0.01	0.01	0.01	0.02	0.01	0.02
Use tabloid media	0.00	0.01	0.02	0.02	0.02	0.02
Trust in the government	0.16^**^	0.05	−0.10	0.07	−0.03	0.08
Trust in science (T1)	0.46^***^	0.06	−0.23^**^	0.09	0.03	0.10
Perceived media exaggeration about COVID-19 (T1)	−0.03	0.03	0.51^***^	0.05	−0.13^*^	0.06
Social distancing behavior (T1)	0.02	0.04	0.03	0.06	0.66^***^	0.10
R^2^	0.51		0.49		0.41	

*N_T1_, 731; N_T2_, 416; T1, Time 1; T2, Time 2*.

* *p < 0.05*,

** *p < 0.01*,

*** *p < 0.001*.

a *dummy-coded variables*.

**Table 3:** Mean values and standard deviation of variables included in the SEM model.

		**T1**			**T2**	
**Variables**	***n***	**Mean**	**SD**	***n***	**Mean**	**SD**
Trust in science	731	3.77	0.88	416	3.57	0.89
Perceived media exaggeration	731	2.51	1.05	416	2.75	1.06
Social distancing behavior	731	3.66	0.99	416	2.85	1.05

We found clear evidence for the reasoning presented in H1 that trust in science decreases perceived media exaggeration over time. That is, trust in science measured at T1 exerted a direct negative effect on perceived media exaggeration about COVID-19 measured at T2 (*b* = −0.23, *SE* = 0.09, *p* = 0.007).

When it comes to H2, we assumed that perceived media exaggeration decreases social distancing behavior over time. Our data supported this expectation. We found that perceived media exaggeration about COVID-19 measured at T1 was a significant negative predictor of COVID-19 related social distancing behavior measured at T2 (*b* = −0.13, *SE* = 0.06, *p* = 0.025).

Additionally, we looked into reciprocal effects. However, we found that media exaggeration about COVID-19 was unrelated to trust in science over time (*b* = −0.03, *SE* = 0.03, *p* = 0.381). Also, social distancing behavior was unrelated to perceived media exaggeration about COVID-19 over time (*b* = 0.03, *SE* = 0.06, *p* = 0.699).

As for control variables, we found a significant effect of our dummy variable indicating the sampling procedure on trust in science measured at T2 (*b* = 0.23, *SE* = 0.07, *p* = 0.001), perceived media exaggeration measured at T2 (*b* = −0.23, *SE* = 0.10, *p* = 0.019) and social distancing behavior measured at T2 (*b* = −0.27, *SE* = 0.11, *p* = 0.014). Further, analysis revealed a significant effect of trust in the government measured at T1 on trust in science measured at T2 (*b* = 0.16, *SE* = 0.05, *p* = 0.001).

## Discussion

The present study was designed to determine the associations between trust in science, perceived media exaggeration, and social distancing behavior during the lockdown in Austria beginning at the end of March and lasting until May 2020. At the time of data collection, people were urged by the government to comply with social distancing behavior due to the uncontrolled outbreak of COVID-19. In the case of Austria, there have been increasing concerns that society becomes more and more divided into two groups: (1) Individuals who fear the virus and engage in the proposed measures and (2) individuals who largely reject scientific evidence, disseminate conspiracy-theory content, and revolt against the new health policies (82). Scientific experts and politicians have been worried that the public's “asymmetrical compliance” with preventative measures could have harmful consequences for the public's health. Although media play a very important role during health crises (7), many individuals perceive the media coverage on pandemics as exaggerated, which may result in information fatigue regarding COVID-19 media coverage (13, 14).

To contribute to this growing area of research, we examined (1) trust in science as an influencing factor on perceptions of media exaggeration about COVID-19, and (2) perceptions of media exaggeration about COVID-19 as a contributing factor to non-compliance with social distancing policies.

Regarding (1) trust in science, we argued that the trustworthiness of the message source may have an impact on how the media coverage about COVID-19 is perceived (54). Since scientists play a major role in media coverage about COVID-19 (1), the level of trust in science could explain why individuals perceive the media as exaggerating the threat of COVID-19. Our assumption was confirmed by the results indicating that trust in science decreased perceptions of media exaggeration about COVID-19. These results are in line with those observed in earlier studies which found that trust in the message source positively correlates with acceptance of the message (83). Moreover, when an expert source is included, messages have more persuasive power over the audience than when no such source is reported (84). Therefore, the results of this study indicate that higher levels of trust in science may make authority cues, such as scientific sources or references to science in media coverage about COVID-19, more salient. As a consequence, this may lead to a spill-over to the audiences' evaluation of the message credibility and thus, to acceptance of the message and a reduction of perceived media exaggeration about COVID-19.

With respect to (2) social distancing behavior in times of COVID-19, we suggested that perceptions of media exaggeration about COVID-19 may influence individuals' compliance with preventative measures. In other words, we argued that perceptions of media exaggeration function as a contributing factor to non-compliance with preventative policies in times of the uncontrolled spread of COVID-19. Drawing on the Theory of Psychological Reactance (16), we assumed that the perception of media exaggeration about COVID-19, as a response to the intense and threatening media coverage of the pandemic, may result in psychological reactance. Consequently, individuals may behave non-conformally with the proposed preventative measures as an attempt to mitigate psychological reactance (16). The current study confirmed this assumption by showing that perceived media exaggeration about COVID-19 negatively influenced social distancing behavior. It is important to note that not only the sole content of the media, but how the media is perceived (i.e., as exaggerated) can have behavioral consequences. This is consistent with other previous research showing that perceptions of media exaggeration of health risks gave rise to non-compliance with preventative measures (13, 72). However, there is room for further progress in predicting social distancing behavior during COVID-19 considering various perceptions about the media or perceptions created by the media in times of health crises. Since, for instance, use of media and perceptions of their truthfulness is positively associated with compliance with social distancing measures, we need to question whether individual factors like risk perceptions of COVID-19 could influence perceptions of media exaggeration too. As trust in science increases risk perceptions, it is most likely that risk perceptions, in turn, decrease perceptions of media exaggeration and thus, function as an additional explanatory factor for our assumed relationship (41, 85, 86). There is, therefore, a definite need for studies taking the role of risk perceptions of COVID-19 into account when investigating trust in science, perceived media exaggeration, and social distancing behavior in times of COVID-19.

Further, it is important to note that we did not observe reversed causality effects for the key variables explored in the present study, suggesting a one-directional relationship between trust in science and perceived media exaggeration about COVID-19 as well as between perceived media exaggeration and social distancing behavior.

The findings of this study make several contributions to the current literature. First, to the best of our knowledge, this is the first study showing that the level of trust in science influences how the media coverage about COVID-19 is perceived. Generally, there is some evidence to suggest that public's trust in science is high (17). However, as the COVID-19 media coverage is very controversial and politicized (1), previous research has shown that the response to COVID-19 scientists is also very politicized. As a consequence, differences in the degree of trust in science between political and sociodemographic groups are developing (19). As this study shows, a certain level of trust in science is involved in the decision whether one accepts media coverage about COVID-19 or perceives it as exaggerated. This finding has equally important practical implications for scientists, journalists, and health communicators, as it highlights the relevance of a trustful source when reporting about unknown health threats. For scientists, it is essential to recognize the importance of public's trust in science during pandemics. They should be aware that they function as an important source of knowledge for people during pandemics. In order to enhance public's trust and thus lead to a higher acceptance of health warnings about COVID-19, scientists should be very careful in communicating their findings in “appealing and transparent ways” characterized by openness and dialogues [(87), p. 13696]. Journalists and health communicators could tackle the issue of audiences' perceptions of media exaggeration about COVID-19 by increasingly referring to trustworthy scientific sources in their articles about COVID-19.

Second, despite the relevance of individuals' perceptions of media coverage in the context of COVID-19, its implications on individuals' social distancing behavior have not been adequately investigated in previous research. This is the first study that has undertaken a longitudinal analysis of the influence of public's perceptions of media exaggeration on social distancing behavior in the context of COVID-19 (41). Understanding the role of the public's perceptions of media coverage of the COVID-19 pandemic in motivating people to engage in social distancing practices may support journalists and health communicators in revising their risk communication strategies. For journalists, this finding has clear implications that underline their great social responsibility in health crises. In the context of the COVID-19 pandemic, journalists should be aware that when their reporting on COVID-19 is perceived as exaggerated, they are contributing to the audiences' ignorance of, or even rebellion against preventative measures like social distancing. Thereby, journalists should increasingly pay attention that they do not contribute to a deterioration of the health crisis situation in times of a pandemic. Thus, we believe that in order to combat COVID-19, journalists must strictly adhere to journalistic standards. Journalists should be very careful to avoid sensationalism and the “obsession to keep churning out breaking news about COVID-19” [(14), p. 266]. Overall, there is a definite need for media coverage about COVID-19 which is characterized by an accurate, fair, and balanced reporting style.

### Limitations and Future Research

The findings of this study are subject to at least seven limitations.

First, the current study was limited by investigating perceived media exaggeration about COVID-19 only in one cultural context. In Austria, the lockdown situation may have been different than in other countries. For instance, some countries have instituted full lockdowns, some have introduced “only” partial lockdowns. Additionally, the extent of punishment of non-compliance with social distancing measures may have been varying among different countries (88). Moreover, we analyzed the associations between trust in science, perceived media exaggeration and social distancing behavior in a country where the media landscape is dominated by a public service organization named ORF that can be categorized as quality media [e.g., (89)]. In the context of COVID-19, the majority of Austrians used information provided by the public broadcasters (including TV program and online news webpage) every day during the COVID-19 crisis. In comparison, tabloid media were used much less to get informed about COVID-19 in these times [i.e., Kronen Zeitung; (90, 91)]. Since the ORF has high journalistic standards, we argue, that the Austrian media coverage was dominated by reporting rather in line with scientific knowledge than display scientists as exaggerating the risks of COVID-19 [e.g., (92)]. However, in other countries like the U.S., media may have taken a stronger partisan perspective with more polarized media coverage of COVID-19 where right-oriented newspapers accuse scientists of exaggerating the crisis [e.g., (1)]. Depending on the media coverage that is dominant, our findings could be different when investigating the associations in different countries. Therefore, the findings of this study may not be generalizable to different countries and populations due to the specificity of the lockdown and the media landscape in Austria. Further work is required to establish comparative results.

Second, data collection took place during the first lockdown. As media coverage of COVID-19 is dynamic (1), the study can only make a statement about the influence of media perceptions on compliance with social distancing behavior at the beginning of the uncontrolled outbreak of COVID-19 under lockdown conditions. Thus, the findings are not generalizable to other lockdowns that have been instituted by the government, nor to those that will occur in the future. Although the time interval between the two waves is justified by the dynamic of the COVID-19 lockdown, with a longer time interval, we would have been able to explore the associations more in depth. Future studies on the current topic in further lockdown contexts are therefore recommended.

Third, the drop-out rate of our panel survey was high (43.1% of participants dropped out) most likely due to the length of the survey^3^. As mentioned in the methods section, we observed a significant difference between drop-outs and non-drop outs for trust in science [*t*_(729)_ = 2.93, *p* = 0.003, d = 0.02]. Although we used Full Maximum Likelihood to estimate the model and thus, account for missing values, and the effect size of the difference is small and the systematic bias would, thus, be negligible, we additionally analyzed the data cross sectionally to be sure that the observed associations are robust including those who dropped out. Results indicated robust findings: Trust in science measured at T1 exerted a direct negative effect on perceived media exaggeration about COVID-19 measured at T1 (*b* = −0.70, *SE* = 0.05, *p* < 0.001). Additionally, media exaggeration about COVID-19 measured at T1 increased social distancing behavior measured at T1 (*b* = −0.15, *SE* = 0.04, *p* < 0.001; CFI = 0.96; TLI = 0.94; NFI = 0.94; χ^2^/df = 2.53, *p* < 0.001; RMSEA = 0.05, 90%-CI [0.04; 0.05]).

Fourth, we measured perceived media exaggeration in terms of the general mainstream media only. Therefore, we cannot make assumptions about the influences of different types of media (e.g., newspapers, TV, radio) exaggeration. In addition, we have not accounted for social media perceptions. It may be that some media are perceived as more exaggerating than other types and may thus serve as a stronger driver for social distancing behavior. Further, perceived media exaggeration was measured in very general terms ignoring the “channels of reporting.” Thus, we cannot make conclusions whether individuals perceive the communicator (e.g., scientists or journalists) or the content (e.g., scientific) as exaggerated. However, this needs to be investigated in future studies.

Fifth, to measure our dependent variable, we used self-reported measures of social distancing. At the time of data collection, participants have been in a lockdown due to COVID-19 which was imposed by the government. Thus, when interpreting the relationship of perceived media exaggeration about COVID-19 and social distancing behavior, one must consider the possible influence of social desirability. Therefore, we point out that the results of the study must be interpreted carefully. By contrast, other studies in the context of COVID-19 have used mobility data to measure compliance with policy measures (2, 60). Further studies, which take objective measures of social distancing behavior into account, will need to be undertaken.

Sixth, we need additional experimental evidence in order to establish that the mechanism of authority cues reduces the perception of media exaggeration. While panel studies allow for examining changes over time, a controlled experimental setting is needed to rule out potential alternative explanations for our findings, for example based on influences that are unrelated to media. Although, in the current study, we controlled for sociodemographics, political orientation, trust in the government as well as media use, other possible influencing factors have not been taken into account due to economic reasons. Further investigation and experimentation into the relationship of trust in science, perceived media exaggeration, and social distancing behavior considering individual heterogeneity is strongly recommended.

Lastly, although we have not hypothesized reciprocal effects, it would be interesting to assess them not only with structural equation modeling like we did, but also with additional approaches like the Granger causality approach (93).

## Conclusion

Non-compliance with social distancing policies could have harmful consequences for the public's health. There is an increasing concern of citizens' “asymmetrical compliance” with these preventative measures to combat COVID-19 (82). Our findings show that trust in science decreased perceived media exaggeration about COVID-19. In turn, the less citizens perceived the media coverage about COVID-19 as exaggerated, the more they reported to act in accordance with social distancing recommendations. Hence, independent of whether the media really exaggerate about the COVID-19 crisis, individuals' sole perception of media exaggeration about COVID-19 can lead to less compliance with governmental measures to combat COVID-19. By and large, our study suggests that media play a role in shaping the course of the COVID-19 health crisis. To conclude, besides implications for journalists and health communicators, this study contributes to the growing effort of researchers to understand the public's (non-) compliance with social distancing measures in times of COVID-19 by exploring media-related factors.

## Data Availability Statement

The datasets presented in this study can be found in online repositories. The names of the repository/repositories and accession number(s) can be found at: https://osf.io/2dsyb/?view_only=71683e3f41de4578a652389c78051dcc.

## Ethics Statement

The studies involving human participants were reviewed and approved by Institutional Review Board of the Department of Communication at the University of Vienna. The patients/participants provided their written informed consent to participate in this study.

## Author Contributions

AN and JM contributed to conception and design of the study. AN organized the data collection and wrote the first draft of the manuscript. JM performed the statistical analysis. MS, RK, and JM wrote sections of the manuscript. All authors contributed to manuscript revision, read, and approved the submitted version.

## Conflict of Interest

The authors declare that the research was conducted in the absence of any commercial or financial relationships that could be construed as a potential conflict of interest.

## Publisher's Note

All claims expressed in this article are solely those of the authors and do not necessarily represent those of their affiliated organizations, or those of the publisher, the editors and the reviewers. Any product that may be evaluated in this article, or claim that may be made by its manufacturer, is not guaranteed or endorsed by the publisher.

## Footnotes

^1^In this paper, we refer to media as “mainstream media” including all major legacy news sources.

^2^We would like to thank the master students of the University of CITY who supported us with the data collection.

^3^The data of this paper was part of a more comprehensive survey project investigating relationships between smartphone use in times of COVID-19 and well-being. Only variables relevant to this paper are reported.

## References

[1] Hart PS Chinn S Soroka S. Politicization and Polarization in COVID-19 News Coverage. Sci Commun. (2020) 42:679–97. 10.1177/1075547020950735 PMC7447862 38602988

[2] Goldstein DAN Wiedemann J. Who Do You Trust? The Consequences of Political and Social Trust for Public Responsiveness to COVID-19 Orders. Cambridge: Cambridge University Press (2020). 10.2139/ssrn.3580547

[3] Haupt F. The Face Mask Moral Decreases. FAZ. Available online at: https://www.faz.net/aktuell/politik/corona-massnahmen-die-mundschutz-moral-broeckelt-16884790.html (accessed November 11, 2021).

[4] TZ. Party With 130 People in Munich. Corona Measures Completely Ignored. (2020). Available online at: https://www.tz.de/muenchen/stadt/coronavirus-muenchen-abstand-maske-party-feier-maskenpflicht-bayern-hygiene-zr-90054296.html (accessed November 11, 2021).

[5] Van Green T Tyson A. 5 Facts About Partisan Reactions to COVID-19 in the U.S. Washington, DC: Pew Research Center (2020). Available online at: https://www.pewresearch.org/facttank/2020/04/02/5-facts-about-partisan-reactions-to-covid-19-in-the-u-s/

[6] Zhang L Kong Y Chang H. Media Use and health behavior in H1N1 Flu crisis: the mediating role of perceived knowledge and fear. Atl J Commun. (2015) 23:67–80. 10.1080/15456870.2015.1013101

[7] Glik DC. Risk communication for public health emergencies. Annu Rev Public Health. (2007) 28:33–54. 10.1146/annurev.publhealth.28.021406.144123 17222081

[8] Kasperson RE Renn O Slovic P Brown HS Emel J Goble R. The social amplification of risk: a conceptual framework. Risk Anal. (1988) 8:177–87. 10.1111/j.1539-6924.1988.tb01168.x

[9] Dudo AD Dahlstrom MF. Brossard D. Reporting a potential pandemic: a risk-related assessment of avian influenza coverage in US newspapers. Sci Commun. (2007) 28:429–54. 10.1177/1075547007302211

[10] Stephens M. A History of News. 3rd Edn. New York, NY: Oxford University Press. (2007).

[11] Vasterman PL Ruigrok N. Pandemic alarm in the Dutch media: media coverage of the 2009 influenza A (H1N1) pandemic and the role of the expert sources. Eur J Commun. (2013) 28:436–53. 10.1177/0267323113486235

[12] Berry TR Wharf-Higgins J Naylor PJ SARS. Wars: an examination of the quantity and construction of health information in the news media. Health Commun. (2007) 21:35–44. 10.1080/10410230701283322 17461750

[13] Rubin GJ Amlot R Page L Wessely S. Public perceptions, anxiety, and behaviour change in relation to the swine flu outbreak: cross sectional telephone survey. BMJ. (2009) 339:1–8. 10.1136/bmj.b2651 19574308 PMC2714687

[14] Ogbodo JN Onwe EC Chukwu J Nwasum CJ Nwakpu ES Nwankwo SU. Communicating health crisis: a content analysis of global media framing of COVID-19. Health Promot Perspect. (2020) 10:257–69. 10.34172/hpp.2020.40 32802763 PMC7420175

[15] Dillard JP Shen L. On the Nature of reactance and its role in persuasive health communication. Commun Monogr. (2005) 72:144–68. 10.1080/0363775050011181533596717

[16] Brehm JW. A Theory of Psychological Reactance. Oxford: Academic Press (1966).

[17] Gallup. Wellcome Global Monitor – First Wave Findings. Technical Report. Wellcome (2019). Available online at: https://wellcome.org/sites/default/files/wellcome-global-monitor-2018.pdf (accessed November 11, 2021).

[18] Petty RE Cacioppo JT. Communication and Persuasion: Central and Peripheral Routes to Attitude Change. Berlin: Springer-Verlag (1986). 10.1007/978-1-4612-4964-1

[19] Evans JH Hargittai E. Who doesn't trust fauci? The public's belief in the expertise and shared values of scientists in the COVID-19 pandemic. Socius Sociol Res Dyn World. (2020) 6:1–13. 10.1177/2378023120947337

[20] Shih T Brossard D Wijaya R. News coverage of public health issues: The role of news sources and the processes of news construction. In: Merrick J, editor. Public Health Yearbook 2011. New York, NY: Nova Science Publishers, Inc. (2011) 93–106.

[21] Hu Y Shyam Sundar S. Effects of online health sources on credibility and behavioral intentions. Commun Res. (2009) 37:105–32. 10.1177/0093650209351512

[22] Glass AJ. The War on Terrorism Goes Online: Media and Government Response to First Post-Internet Crisis [Internet] (2002). p. 28. Available online at: https://shorensteincenter.org/wp-content/uploads/2012/03/2002_03_glass.pdf (accessed November 11, 2021).

[23] Brug J Aro AR Oenema A de Zwart O Richardus JH Bishop GD. Risk perception, knowledge, precautions, and information sources, the Netherlands. Emerg Infect Dis. (2004) 10:1486–9. 10.3201/eid1008.040283 15496256 PMC3320399

[24] Washer P. Representations of SARS in the British newspapers. Soc Sci Med. (2004) 59:2561–71. 10.1016/j.socscimed.2004.03.038 15474209 PMC7127057

[25] Yu N Frohlich DO Fougner J Ren L. Communicating a health epidemic: a risk assessment of the swine flu coverage in US newspapers. Int Public Health J. (2011) 3:63–76.

[26] Mejia CR Ticona D Rodriguez-Alarcon JF Campos-Urbina AM Catay-Medina JB Porta-Quinto T. The media and their informative role in the face of the Coronavirus Disease 2019 (COVID-19): validation of fear perception and magnitude of the issue (MED-COVID-19). Electron J Gen Med. (2020) 17:em239. 10.29333/ejgm/79467946

[27] Lewison G. The reporting of the risks from severe acute respiratory syndrome (SARS) in the news media, 2003–2004. Health Risk Soc. (2008) 10:241–62. 10.1080/13698570802160962

[28] Ungar S. Global bird flu communication: hot crisis and media reassurance. Sci Commun. (2008) 29:472–97. 10.1177/1075547008316219

[29] Klemm C Das E Hartmann T. Swine flu and hype: a systematic review of media dramatization of the H1N1 influenza pandemic. J Risk Res. (2016) 19:1–20. 10.1080/13669877.2014.923029

[30] Bennett WL. The burglar alarm that just keeps ringing: a response to Zaller. Polit Commun. (2003) 20:131–8. 10.1080/10584600390211145

[31] McCright AM Dentzman K Charters M Dietz T. The influence of political ideology on trust in science. Environ Res Lett. (2013) 8:044029. 10.1088/1748-9326/8/4/044029

[32] Kritzinger S Foucault M Lachat R Partheymüller J Plescia C Brouard S. ‘Rally round the flag': the COVID-19 crisis and trust in the national government. West Eur Polit. (2021) 44:1205–31. 10.1080/01402382.2021.1925017

[33] Sibley CG Greaves LM Satherley N Wilson MS Overall NC Lee CHJ. Effects of the COVID-19 pandemic and nationwide lockdown on trust, attitudes toward government, and well-being. Am Psychol. (2020) 75:618–30. 10.1037/amp0000662 32496074

[34] Bavel JJV Baicker K Boggio PS Capraro V Cichocka A Cikara M. Using social and behavioural science to support COVID-19 pandemic response. Nat Hum Behav. (2020) 4:460–71. 10.1038/s41562-020-0884-z 32355299

[35] Roozenbeek J Schneider CR Dryhurst S Kerr J Freeman ALJ Recchia G. Susceptibility to misinformation about COVID-19 around the world. R Soc Open Sci. (2020) 7:1–15. 10.1098/rsos.201199 33204475 PMC7657933

[36] Chan HF Rizio SM Skali A Torgler B. Early COVID-19 government communication is associated with reduced interest in the QAnon conspiracy theory. Front Psychol. (2021) 12:681975. 10.3389/fpsyg.2021.681975 34531787 PMC8438198

[37] Bargain O Aminjonov U. Trust and compliance to public health policies in times of COVID-19. J Public Econ. (2020) 192:104316. 10.1016/j.jpubeco.2020.104316 33162621 PMC7598751

[38] Dohle S Wingen T Schreiber M. Acceptance and adoption of protective measures during the COVID-19 pandemic: the role of trust in politics and trust in science. Soc Psychol Bull. (2020) 15:e4315. 10.32872/spb.4315

[39] Pagliaro S Sacchi S Pacilli MG Brambilla M Lionetti F Bettache K. Trust predicts COVID-19 prescribed and discretionary behavioral intentions in 23 countries. PLoS One. (2021) 16:e0248334. 10.1371/journal.pone.0248334 33690672 PMC7946319

[40] Wu Y Shen F. Exploring the impacts of media use and media trust on health behaviors during the COVID-19 pandemic in China. J Health Psychol. (2021) 1–17. 10.1177/1359105321995964 33646827

[41] Devine D Gaskell J Jennings W Stoker G. Trust and the coronavirus pandemic: what are the consequences of and for trust? An early review of the literature. Polit Stud Rev. (2020) 19:274–85. 10.1177/1478929920948684 PMC7424609 35082554

[42] Zafri NM Afroj S Nafi IM. Hasan MdMU. A content analysis of newspaper coverage of COVID-19 pandemic for developing a pandemic management framework. Heliyon. (2021) 7:e06544. 10.1016/j.heliyon.2021.e06544 33758786 PMC7973064

[43] Aranzales I Chan HF Eichenberger R Hegselmann R Stadelmann D Torgler B. Scientists have favorable opinions on immunity certificates but raise concerns regarding fairness and inequality. Sci Rep. (2021) 11:14016. 10.1038/s41598-021-93148-1 34234190 PMC8263576

[44] Barke RP Jenkins-Smith HC. Politics and scientific expertise: scientists, risk perception, and nuclear waste policy. Risk Anal. (1993) 13:425–39. 10.1111/j.1539-6924.1993.tb00743.x 8234951

[45] Wormer H. German media and coronavirus: exceptional communication—or just a catalyst for existing tendencies? Media Commun. (2020) 8:467–70. 10.17645/mac.v8i2.3242

[46] Battiston P Kashyap R Rotondi V. Trust in Science and Experts During the COVID-19 Outbreak in Italy. SocArXiv. (2020). 10.31235/osf.io/5tch8 33553567 PMC7859315

[47] Siegrist M Cvetkovich G. Perception of hazards: the role of social trust and knowledge. Risk Anal. (2000) 20:713–20. 10.1111/0272-4332.205064 11110217

[48] Stadelmann D Torgler B. Bounded rationality and voting decisions over 160 years: voter behavior and increasing complexity in decision-making. PLoS ONE. (2013) 8:e84078. 10.1371/journal.pone.0084078 24391888 PMC3877213

[49] Lebernegg NS Eberl J-M Boomgaarde HG Partheymüller J. Alte und neue Medien: Informationsverhalten in Zeiten der Corona-Krise [Old and New Media: Information Behavior in Times of the Corona Crisis]. Austrian Corona Panel Project: Corona-Blog. (2020). Available online at: https://viecer.univie.ac.at/corona-blog/corona-blog-beitraege/blog04/ (accessed November 11, 2021).

[50] Fischeneder A Balluff P Boomgaarden H Eisele O Litvyak O Brändle VK. Berichterstattung über die COVID-19 Krise in Standard und Krone [News reporting on the COVID-19 crisis in Standard and Krone]. Austrian Corona Panel Project: Corona-Blog [Internet]. (2020). Available online at: https://viecer.univie.ac.at/corona-blog/corona-blog-beitraege/blog92/ (accessed November 11, 2021).

[51] Hanitzsch T Van Dalen A Steindl N. Caught in the Nexus: a comparative and longitudinal analysis of public trust in the press. Int J Press. (2018) 23:3–23. 10.1177/1940161217740695

[52] Earl Bennett S Rhine SL Flickinger RS Bennett LLM. “Video Malaise” revisited: public trust in the media and government. Harv Int J Press. (1999) 4:8–23. 10.1177/1081180X9900400402

[53] Stecula DA Pickup M. How populism and conservative media fuel conspiracy beliefs about COVID-19 and what it means for COVID-19 behaviors. Res Polit. (2021) 8:205316802199397. 10.1177/2053168021993979

[54] Slater MD Rouner D. How message evaluation and source attributes may influence credibility assessment and belief change. J Mass Commun Q. (1996) 73:974–91. 10.1177/107769909607300415

[55] Merkley E. Anti-intellectualism, populism, and motivated resistance to expert consensus. Public Opin Q. (2020) 84:24–48. 10.1093/poq/nfz053

[56] World Health Organization. Coronavirus Disease (COVID-19) Advice for the Public. (2020). Available online at: https://www.who.int/emergencies/diseases/novel-coronavirus-2019/advice-for-public (accessed October 1, 2021).

[57] Meyer HK Marchionni D. Thorson E. The journalist behind the news: credibility of straight, collaborative, opinionated, and blogged “News.” Am Behav Sci. (2010) 54:100–19. 10.1177/0002764210376313

[58] Heider F. The Psychology of Interpersonal Relations. Hoboken, NJ: John Wiley & Sons Inc. (1959).

[59] Bierwiaczonek K Kunst JR Pich O. Belief in COVID-19 conspiracy theories reduces social distancing over time. Appl Psychol Health Well-Being. (2020) 12:1270–85. 10.1111/aphw.12223 32864837

[60] Valenti VE Menezes P de L Abreu ACG de Vieira GNA Garner DM. Social distancing measures may have reduced the estimated deaths related to Covid-19 in Brazil. J Hum Growth Dev. (2020) 30:164–9. 10.7322/jhgd.v30.10360

[61] Park S Kim B Lee J. Social distancing and outdoor physical activity during the COVID-19 outbreak in South Korea: implications for physical distancing strategies. Asia Pac J Public Health. (2020) 32:360–2. 10.1177/1010539520940929 32667221 PMC7364329

[62] Marroquín B Vine V Morgan R. Mental health during the COVID-19 pandemic: effects of stay-at-home policies, social distancing behavior, and social resources. Psychiatry Res. (2020) 293:113419. 10.1016/j.psychres.2020.113419 32861098 PMC7439968

[63] News. Lockdown in Austria. A Chronology. (2020). Available online at: https://www.news.at/a/coronavirus—lockdown-im-fruehling-lockdown-light-im-herbst-1-11729350

[64] Nielsen RK Fletcher R Kalogeropoulos A Simon F. Communications in the Coronavirus Crisis: Lessons for the Second Wave. Oxford: Reuters Institute for the Study of Journalism (2020). Available online at: https://reutersinstitute.politics.ox.ac.uk/communications-coronavirus-crisis-lessons-second-wave (accessed November 11, 2021).

[65] Bridgman A Merkley E Loewen PJ Owen T Ruths D Teichmann L. The causes and consequences of COVID-19 misperceptions: Understanding the role of news and social media. Harv Kennedy Sch Misinformation Rev. (2020) 1:1–18. 10.37016/mr-2020-028

[66] Nwakpu ES Ezema VO Ogbodo JN. Nigeria media framing of coronavirus pandemic and audience response. Health Promot Perspect. (2020) 10:192–9. 10.34172/hpp.2020.32 32802755 PMC7420160

[67] Sandell T Sebar B Harris N. Framing risk: Communication messages in the Australian and Swedish print media surrounding the 2009 H1N1 pandemic. Scand J Public Health. (2013) 41:860–5. 10.1177/1403494813498158 23873631

[68] Mutua SN. Ong'ong'a DO. Online news media framing of COVID-19 pandemic: probing the initial phases of the disease outbreak in international media. Eur J Interact Multimed Educ. (2020) 1:e02006. 10.30935/ejimed/8402

[69] Witte K. Putting the fear back into fear appeals: the extended parallel process model. Commun Monogr. (1992) 59:329–49. 10.1080/0363775920937627623330860

[70] Bensley LS Wu R. The role of psychological reactance in drinking following alcohol prevention messages1. J Appl Soc Psychol. (1991) 21:1111–24. 10.1111/j.1559-1816.1991.tb00461.x

[71] Vasterman PLM. Media-hype: self-reinforcing news waves, journalistic standards and the construction of social problems. Eur J Commun. (2005) 20:508–30. 10.1177/0267323105058254

[72] Prati G Pietrantoni L Zani B. Compliance with recommendations for pandemic influenza H1N1 2009: the role of trust and personal beliefs. Health Educ Res. (2011) 26:761–9. 10.1093/her/cyr035 21613380

[73] Taris TW Kompier MAJ. Cause and effect: optimizing the designs of longitudinal studies in occupational health psychology. Work Stress. (2014) 28:1–8. 10.1080/02678373.2014.878494

[74] Punz H. Die Chronologie des Corona-Lockdowns. Die Presse. (2021). Available online at: https://www.diepresse.com/5924014/die-chronologie-der-corona-lockdowns (accessed November 11, 2021).

[75] Der Standard. Police Dispersed Unauthorized Demo in Vienna. (2020). Available online at: https://www.derstandard.at/story/2000117100193/trotz-verbots-fand-kundgebung-gegen-corona-massnahmen-in-wien-statt (accessed November 11, 2021).

[76] Łaszewska A Helter T Simon J. Perceptions of Covid-19 lockdowns and related public health measures in Austria: a longitudinal online survey. BMC Public Health. (2021) 21:1502. 10.1186/s12889-021-11476-3 34344343 PMC8331215

[77] Traunmüller C Stefitz R Gaisbachgrabner K Schwerdtfeger A. Psychological correlates of COVID-19 pandemic in the Austrian population. BMC Public Health. (2020) 20:1395. 10.1186/s12889-020-09489-5 32928180 PMC7487438

[78] Pieh C Budimir S Probst T. The effect of age, gender, income, work, and physical activity on mental health during coronavirus disease (COVID-19) lockdown in Austria. J Psychosom Res. (2020) 136:110186. 10.1016/j.jpsychores.2020.110186 32682159 PMC7832650

[79] AGES. AGES Dashboard COVID19. (2021). Available online at: https://covid19-dashboard.ages.at/dashboard.html (accessed November 11, 2021).

[80] Arbuckle JL. Amos (version 23.0) [Computer Program]. Chicago: IBM SpSS.

[81] Vandenberg RJ Lance CE. A review and synthesis of the measurement invariance literature: suggestions, practices, and recommendations for organizational research. Organ Res Methods. (2000) 3:4–70. 10.1177/109442810031002

[82] Der Standard. Around 1,500 People Demonstrated Against Corona Measures in Vienna Without Distance and Without a Face Mask. (2020). Available online at: https://www.derstandard.at/story/2000121201027/rund-1-500-corona-verharmloser-demonstrieren-in-wien-ohne-abstand (accessed November 11, 2021).

[83] Poortinga W Pidgeon NF. Trust in risk regulation: cause or consequence of the acceptability of GM food? Risk Anal. (2005) 25:199–209. 10.1111/j.0272-4332.2005.00579.x 15787769

[84] Andersen K Clevenger T. A summary of experimental research in ethos. Speech Monogr. (1963) 30:59–78. 10.1080/03637756309375361

[85] Schneider CR Dryhurst S Kerr J Freeman ALJ Recchia G Spiegelhalter D. COVID-19 risk perception: a longitudinal analysis of its predictors and associations with health protective behaviours in the United Kingdom. J Risk Res. (2021) 24:294–313. 10.1080/13669877.2021.1890637

[86] Vai B Cazzetta S Ghiglino D Parenti L Saibene G Toti M. Risk perception and media in shaping protective behaviors: insights from the early phase of COVID-19 Italian outbreak. Front Psychol. (2020) 11:563426–563426. 10.3389/fpsyg.2020.563426 33250809 PMC7674945

[87] Boele-Woelki K Francisco JS Hahn U Herz J. How we can rebuild trust in science—and why we must. Angew Chem Int Ed. (2018) 57:13696–7. 10.1002/anie.201805342 30039913

[88] Dunford D Dale B Stylianou N Lowther E Ahmed M De la Torre Arenas I. Coronavirus: The World in Lockdown in Maps and Charts. BBC. (2020). Available online at: https://www.bbc.com/news/world-52103747 (accessed November 11, 2021).

[89] Karmasin M Kraus D Kaltenbrunner A Bichler K. Austria: A border-crosser. In: Eberwein T, Fengler S, Lauk E, Leppik-Bork T, editors. Mapping Media Accountability—in Europe and Beyond. Köln: Halem Verlag (2011). p. 22–35.

[90] Eberl J-M Lebernegg NS. Die alternativen COVID-Realitäten des österreichischen TV-Publikums [The Alternative COVID Realities of the Austrian TV Audience]. Austrian Corona Panel Project: Corona-Blog. (2021). Available online at: https://viecer.univie.ac.at/en/projects-and-cooperations/austrian-corona-panel-project/corona-blog/corona-blog-beitraege/blog125/ (accessed November 11, 2021).

[91] Moosbrugger R Prandner D Glatz C. Soziale Ungleichheiten im Informationsverhalten während der Corona Krise [Social Inequalities in Information Seeking Behavior During the Corona Crisis]. Austrian Corona Panel Project: Corona-Blog. (2020). Available online at: https://viecer.univie.ac.at/en/projects-and-cooperations/austrian-corona-panel-project/corona-blog/corona-blog-beitraege/blog55/ (accessed November 11, 2021).

[92] Stark B. Qualitätsmedien und ihr Publikum in Zeiten des Medienwandels – das Fallbeispiel ORF. In: Gonser N, editor. Die multimediale Zukunft des Qualitätsjournalismus. New York: Springer VS (2013). p. 53–67.

[93] Granger CWJ. Investigating causal relations by econometric models and cross-spectral methods. Econometrica. (1969) 37:424–38. 10.2307/1912791

